# Electrical Characteristics of CMOS-Compatible SiO*_x_*-Based Resistive-Switching Devices

**DOI:** 10.3390/nano13142082

**Published:** 2023-07-16

**Authors:** Maria N. Koryazhkina, Dmitry O. Filatov, Stanislav V. Tikhov, Alexey I. Belov, Dmitry A. Serov, Ruslan N. Kryukov, Sergey Yu. Zubkov, Vladislav A. Vorontsov, Dmitry A. Pavlov, Evgeny G. Gryaznov, Elena S. Orlova, Sergey A. Shchanikov, Alexey N. Mikhaylov, Sungjun Kim

**Affiliations:** 1 Research and Education Center “Physics of Solid-State Nanostructures”, National Research Lobachevsky State University of Nizhny Novgorod, 603022 Nizhny Novgorod, Russia; 2 Department of English for Natural Sciences, National Research Lobachevsky State University of Nizhny Novgorod, 603022 Nizhny Novgorod, Russia; 3 Institute of Nanotechnologies Electronics and Equipment Engineering, Southern Federal University, 347922 Taganrog, Russia; 4 Department of Information Technologies, Vladimir State University, 600000 Vladimir, Russia; 5 Division of Electronics and Electrical Engineering, Dongguk University, Seoul 04620, Republic of Korea

**Keywords:** memristor, resistive switching, silicon oxide, electrical characteristics, thermal treatment

## Abstract

The electrical characteristics and resistive switching properties of memristive devices have been studied in a wide temperature range. The insulator and electrode materials of these devices (silicon oxide and titanium nitride, respectively) are fully compatible with conventional complementary metal-oxide-semiconductor (CMOS) fabrication processes. Silicon oxide is also obtained through the low-temperature chemical vapor deposition method. It is revealed that the as-fabricated devices do not require electroforming but their resistance state cannot be stored before thermal treatment. After the thermal treatment, the devices exhibit bipolar-type resistive switching with synaptic behavior. The conduction mechanisms in the device stack are associated with the effect of traps in the insulator, which form filaments in the places where the electric field is concentrated. The filaments shortcut the capacitance of the stack to different degrees in the high-resistance state (HRS) and in the low-resistance state (LRS). As a result, the electron transport possesses an activation nature with relatively low values of activation energy in an HRS. On the contrary, Ohm’s law and tunneling are observed in an LRS. CMOS-compatible materials and low-temperature fabrication techniques enable the easy integration of the studied resistive-switching devices with traditional analog–digital circuits to implement new-generation hardware neuromorphic systems.

## 1. Introduction

At present, artificial intelligence (AI) systems play a significant role in our everyday life. At the same time, more and more complex AI applications require the development of efficient neuromorphic (brain-inspired) computing technologies [1]. New AI hardware is subject to a number of requirements for compactness, energy efficiency, and compatibility with the conventional complementary metal-oxide-semiconductor (CMOS) fabrication process.

A memristor [2] or a memristive device is a simple two-terminal electronic device, which is able to change its resistive state adaptively depending on the voltage applied and maintain it for a long time. The change in resistance (resistive switching between at least two states: a low-resistance state (LRS) and a high-resistance state (HRS)) usually occurs due to rupturing and restoring the conductive filaments inside the insulator film sandwiched between metal electrodes. The diameter of filaments can range from 1 to 100 nm depending on the materials used and the mechanism behind their formation [3].

Resistive-switching devices are suitable for applications as compact (nanometer-sized) and energy-efficient (femtojoules per switching operation) Resistive Random-Access Memory devices, which can be easily integrated back-end-of-line (BEOL) with the front-end-of-line (FEOL) CMOS circuitry [4]. Such devices are capable of not only storing the Boolean values encoded by the resistance values (LRS or HRS), but also allow the alteration of these ones inside the memory circuits implementing the so-called “non-von Neumann” or “in-memory computing” paradigm. In addition, a simple structure of the memristive device stack allows for the development of super-dense and potentially three-dimensional cross-bar arrays, which implement the vector–matrix multiplication operations underlying inference in traditional deep learning artificial neural networks [5,6] and new learning algorithms for training the spiking neural networks [7].

In the present work, the electrical characteristics and resistive switching properties of metal–insulator–metal (MIM) device stacks based on a SiO*_x_* film obtained through a low-temperature plasma-enhanced chemical vapor deposition (PECVD) method were studied with the final goal of integrating these stacks into modern CMOS circuits.

## 2. Materials and Methods

The SiO*_x_* films with a nominal thickness of 13 nm were deposited onto standard factory-made TiN(25 nm)/Ti(25 nm)/SiO_2_(500 nm)/Si(001) substrates using the PECVD method. The parameters of the deposition process were described elsewhere [8]. Next, the Au (20 nm) top electrodes with Zr sublayers (8 nm) were deposited onto the SiO*_x_* films by direct-current (DC) magnetron sputtering at the substrate temperature 200 °C. The areas of the top electrodes were *S* ~ 10^−2^ cm^2^. The metallized substrate served as the bottom electrode.

The SiO*_x_* films after the deposition onto the substrates were studied using X-ray photoelectron spectroscopy (XPS) with Ar^+^ ion sputtering (the ion energy was 2 keV) using the Omicron^®^ MultiProbe™ RM ultra-high vacuum (UHV) (Taunusstein, Germany) system to determine the depth distribution of species and their chemical state. The structural investigations of the memristive stacks after thermal treatment were carried out using high-resolution cross-sectional transmission electron microscopy (HR X-TEM) using the Jeol^®^ 2100F microscope (Tokyo, Japan). The electron accelerating voltage was 180 keV.

The electrical characteristics and resistive switching parameters were studied using the Agilent^®^ B1500A semiconductor device parameter analyzer (Santa Rosa, CA, USA). The sign of the voltage applied to memristive devices corresponded to the potential of the top electrode (Au) relative to the potential of the bottom electrode (TiN). In the present study, the current–voltage (*I–V*) curves of memristive devices, as well as the small-signal frequency (*f*) dependencies of the capacitance *C*(*f*), of the conductance *G*(*f*), and of the resistance *R*(*f*) for the parallel and serial resistor–capacitor equivalent circuits, were studied in the LRS and in the HRS at different temperatures, as in [8]. The capacitance–voltage (*C–V*) and conductance–voltage (*G–V*) curves were also investigated. During measurements, the instrumental error did not exceed ±0.92% for *C*, ±99 μS for *G*, ±10% for *V*, ±0.008% for *f*, and ±1% for *I*. The specified instrument errors corresponded to those from the equipment user manual for the Agilent^®^ B1500A semiconductor device parameter analyzer (Santa Rosa, CA, USA).

## 3. Results and Discussion

**Figure 1:**
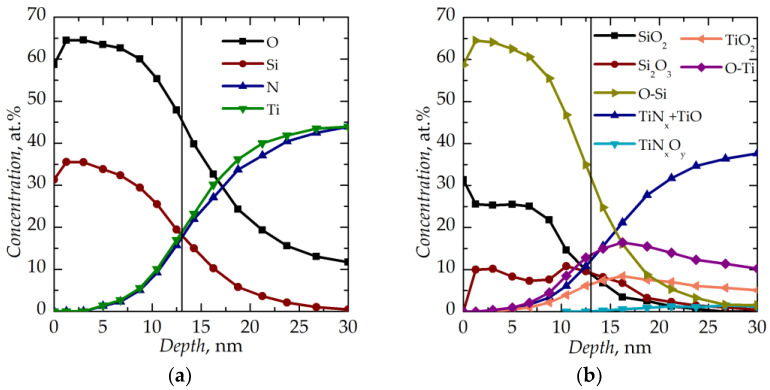
Depth distributions (**a**) of species and (**b**) of their chemical state in the SiO*_x_* film on the metallized substrate. The vertical lines show the interfaces between the SiO*_x_* film and the substrate.

The results of the XPS characterization of the initial SiO*_x_* film on the substrate are shown in Figure 1. One can see the oxygen fraction *x* in the SiO*_x_* film material varying from 1.8–1.9 at the film surface up to 2.5–2.6 at the interface with the substrate corresponding to various silicon oxide types revealed in the chemical composition of the film. The partial oxidation of the titanium nitride bottom electrode should also be noted.

The measurements of the frequency dependencies of the equivalent circuit parameters of the memristive stack in the initial state demonstrate high Ohmic losses (up to tg*δ* ~ 30 at *f* = 1 kHz), a high value of dielectric permittivity *ε* ≈ 9.8 at *f* = 100 kHz, and a low value of parallel resistance *R_p_* ~ 10 kΩ at *f* = 1 kHz. The above values can be explained by the presence of conductive defects nonuniformly distributed in the insulator in the initial state. The localization of such defects in the form of conductive channels similar to filaments during the deposition of the top electrodes can probably be facilitated by the contact potential difference between the top electrode and the bottom one. This is due to the different values of the electron work function for Zr (4.05 eV [9]) and for TiN (4.3–4.65 eV [10]), as well as the low thickness of the SiO*_x_* film. As a result, the electric field strength in the insulator at a zero bias is high enough ((1.25–3) × 10^5^ V/cm) to promote the filament formation.

**Figure 2:**
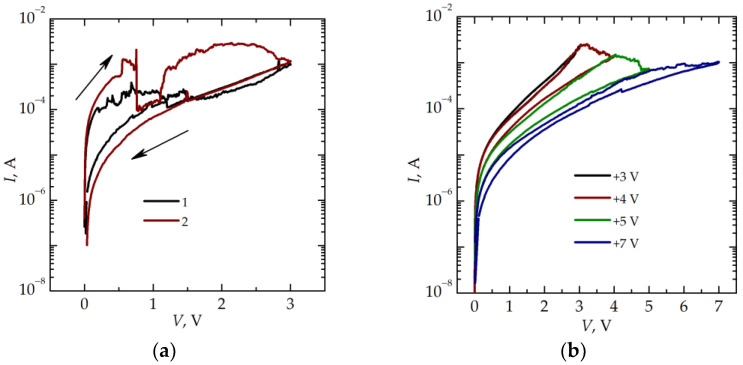
(**a**) *I–V* curves of the SiO*_x_*-based memristive stack in the initial state (curve 2 is measured several minutes after curve 1). The directions of the voltage sweep are shown by arrows. (**b**) *I–V* curves of the memristive device in multiple HRSs after thermal treatment.

Figure 2a shows the *I*–*V* curves of the memristive stack in the initial state. Under an applied voltage of +3 V, the stack switches from the initial LRS to an HRS (Figure 2a, curve 1). Repeated measurements of the *I–V* curves in the same conditions demonstrate the LRS is restored after few minutes (Figure 2a, curve 2).

Earlier, the thermal treatment of memristive stacks was shown to result in an irreversible change in their electrical characteristics and to positively affect the resistive switching performance of these stacks positively in some cases [8]. Because the memristive stacks under study did not demonstrate stable resistive switching in the initial state, they were subjected to thermal treatment in dried air ambient in a sealed thermostat at 250 °C for 10 min. This results in a change in the nature of resistive switching and in an irreversible change in electrical characteristics.

After the thermal treatment, the devices demonstrate a decrease in the dielectric losses at low frequencies, in the leakage currents, and in the *ε* value (down to ≈ 5.4 at *f* = 100 kHz). These changes are associated with the oxidation of excess Si during the thermal treatment. After this treatment, the stacks also demonstrate bipolar resistive switching with synaptic behavior (Figure 2b). Namely, the current values in multiple HRSs decrease gradually with increasing voltage.

**Figure 3:**
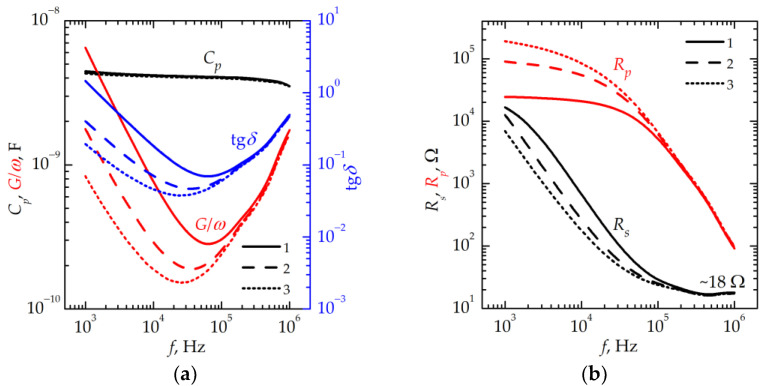
Frequency dependencies (**a**) of *C_p_*, *G/ω*, and tg*δ*, and (**b**) of *R_s_* and *R_p_* for the memristive device after the successive application of the voltage of +3 V (curves 1), +5 V (curves 2), and +7 V (curves 3).

Figure 3 shows the frequency dependencies of the equivalent circuit parameters of a memristive device in the HRS obtained after the successive application of a positive voltage (Figure 2b). An increase in the voltage applied leads to a change in the equivalent circuit parameters in the frequency range *f* < 10^5^ Hz. The increase in dielectric losses with a decreasing frequency is described by losses of the Ohmic nature [11], which are minimal after switching with a voltage of +7 V. In the same frequency range, *R_p_* decreases with increasing voltage. However, the essential shortcutting effect of the Ohmic leakage is also evident after switching with the voltage of +7 V. Thus, it is not possible to eliminate the effect of filaments on the electrical characteristics in the stack under study completely. It should be noted that the synaptic behavior revealed in the gradual resistance change also takes place under the negative voltage, which transfers the device to an LRS. In this case, the changes in the value of *R_p_* and in the dielectric losses in the low-frequency range also occur. It is important to note that, in both cases (HRS and LRS), there are no changes in *R_s_* at high frequencies. At *f* = 1 MHz, *R_s_* is ~18 Ω, the value of *R_s_* at a high frequency is determined by the resistance of the top electrode [12]. The absence of changes in *R_s_* at high frequencies indicates the absence of electrochemical processes leading to a change in resistance in the top electrode.

**Figure 4:**
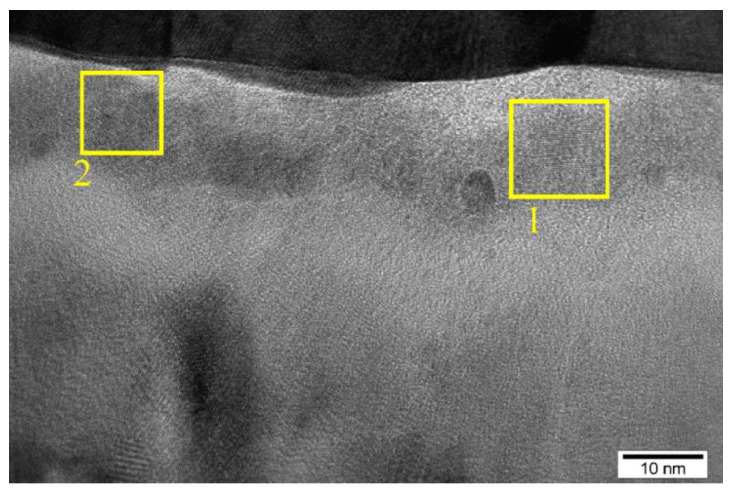
The HR X-TEM image of a SiO*_x_*-based memristive stack after thermal treatment.

Figure 4 shows an HR X-TEM image of a SiO*_x_*-based memristive device after the thermal treatment. According to Figure 4, the SiO*_x_* film has an amorphous structure with nanocrystals identified as Zr_3_O (area 1) and ZrO_2_ (area 2) through a comparison of the interplanar spacings in the HR X-TEM image with the literature data performed earlier [8]. Such nanocrystals can form during thermal treatment due to a partial oxidation of the top electrode layer and can play a positive role in the localization of conductive filaments, e.g., as electric field concentrators [13]. Note that a link between the HR X-TEM results and the electrical characteristics can be obtained in more detail by using a combined approach, for example using the first-principle calculation and electron energy-loss spectroscopy. This was performed by the authors of [14], who showed that oxygen in Bi_2_S_3_ nano-networks is a source of traps. Moreover, the authors note that continuously distributed traps are responsible for the gradual resistive switching (i.e., with a synaptic behavior), while the presence of discrete states leads to the abrupt resistive switching.

**Figure 5:**
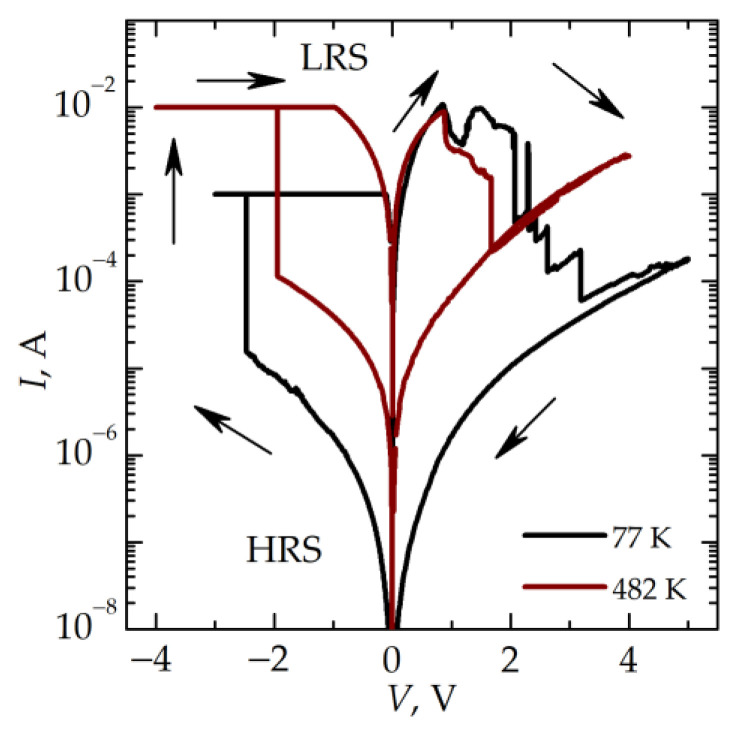
*I–V* curves of the memristive device after the thermal treatment measured at different temperatures. The directions of the voltage sweep are shown by arrows.

In order to determine the mechanisms of electron transport in the memristive device, the electrical characteristics and resistive switching are considered in a wide temperature range. Figure 5 shows the *I–V* curves of the memristive device measured at 77 and 482 K. The *I–V* curves in the LRS obey Ohm’s law. In an HRS, these curves are nonlinear. The current in an LRS exceeds the current in an HRS by two orders of magnitude at 482 K and by four orders of magnitude at 77 K.

**Figure 6:**
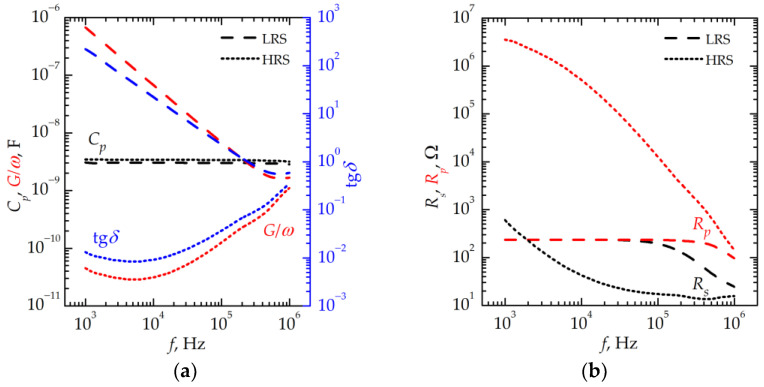
Frequency dependencies (**a**) of *C_p_*, *G/ω*, and tg*δ*, and (**b**) of *R_s_* and *R_p_* for the memristive device in LRS and in HRS at 77 K.

**Figure 7:**
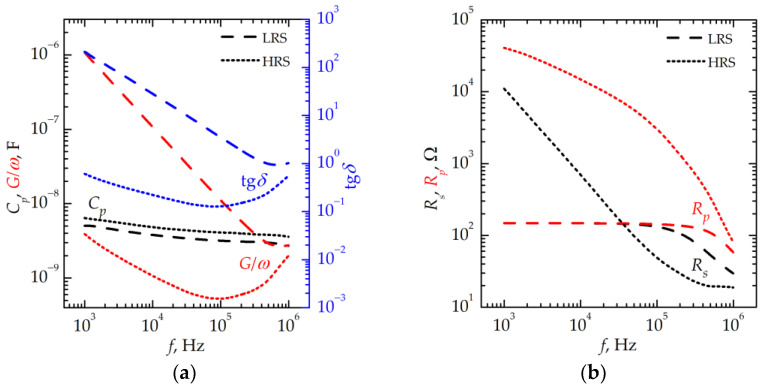
Frequency dependencies (**a**) of *C_p_*, *G/ω*, and tg*δ*, and (**b**) of *R_s_* and *R_p_* for the memristive device in LRS and in HRS at 482 K.

Figure 6 and Figure 7 show the frequency dependencies of the equivalent circuit parameters of the memristive device after the thermal treatment obtained after measuring the *I–V* curves shown in Figure 5.

At 77 K, almost no effect of the filaments on the equivalent circuit parameters of the device in an HRS is found in the low-frequency range. In this range, there are almost no dielectric losses associated with the Ohmic leakage. It probably may originate from the mechanical strain arising during cooling down the stack. Similar phenomena and the thermal resistive switching have been previously found in HfO_x_-based memristive devices [15]. The value of *R_p_* shunting the stack in the HRS was ~3.5 × 10^6^ Ω at *f* = 1 kHz. After switching to the LRS, the value of *R_p_* decreases down to ~200 Ω, whereas the Ohmic losses increase due to the restoration of filaments. The value of *R_s_* at a high frequency, which is determined by the resistance of the top electrode, almost does not change during switching.

The equivalent circuit parameters of the memristive device in an LRS at 482 K almost do not differ from those measured at 77 K. However, the low-frequency dielectric losses remain due to the presence of incompletely broken filaments in an HRS.

**Figure 8:**
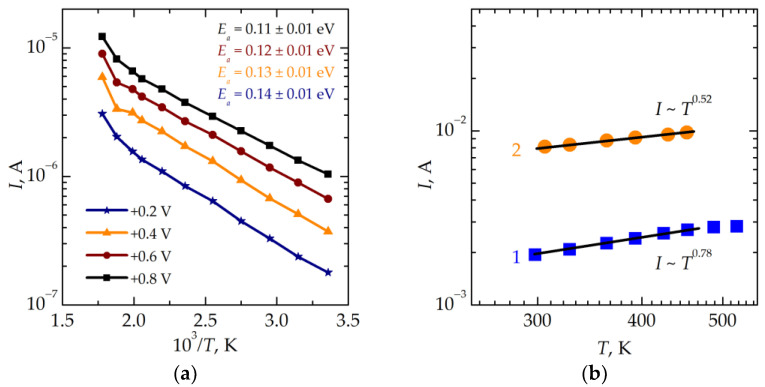
(**a**) Temperature dependencies of the current flowing through the memristive device in an HRS measured at different reading voltages. (**b**) Temperature dependencies of the current flowing through the memristive device in an LRS measured at the reading voltage of +0.1 V (curve 1 was measured after switching to an LRS by applying a voltage of −4 V; curve 2—by applying a voltage of −7 V).

Figure 8a shows the temperature dependencies of the current flowing through the device in an HRS measured in the temperature range from 300 to 560 K at different reading voltages. One can see that these dependencies have an activation character and can be fitted by straight lines in the lg*I*(1/*T*) scales in the temperature range from 300 to 530 K. Moreover, the values of activation energy *E_a_* determined from the slopes of the fitting lines decrease with increasing reading voltages from 0.14 eV to 0.11 eV. The linear extrapolation of the dependence *E_a_*(*V*^1/2^) to zero gives a value of 0.15 eV. The character of the above dependences in an HRS can indicate the Schottky electron transport mechanism or the Poole–Frenkel one [16]. However, low values of activation energy can be attributed to the presence of filaments shunting the functional insulator layer. The impact of filaments cannot be excluded completely in the temperature range investigated.

In an LRS, the nonlinear character of conduction remains even at low resistances (12.5 Ω at the room temperature, curve 2 in Figure 8b). The conductivity is characterized by a power-law temperature dependence of the current with a small exponent (<1) depending on the resistance of the sample and, therefore, on the mode of switching from an HRS to an LRS. Such a weak temperature dependence of the current points to the trap-assisted tunneling electron transport mechanism [17,18]. It should be stressed here that the electron transport in an LRS is confined predominantly within a thin filament with a diameter down to ~1 nm.

**Figure 9:**
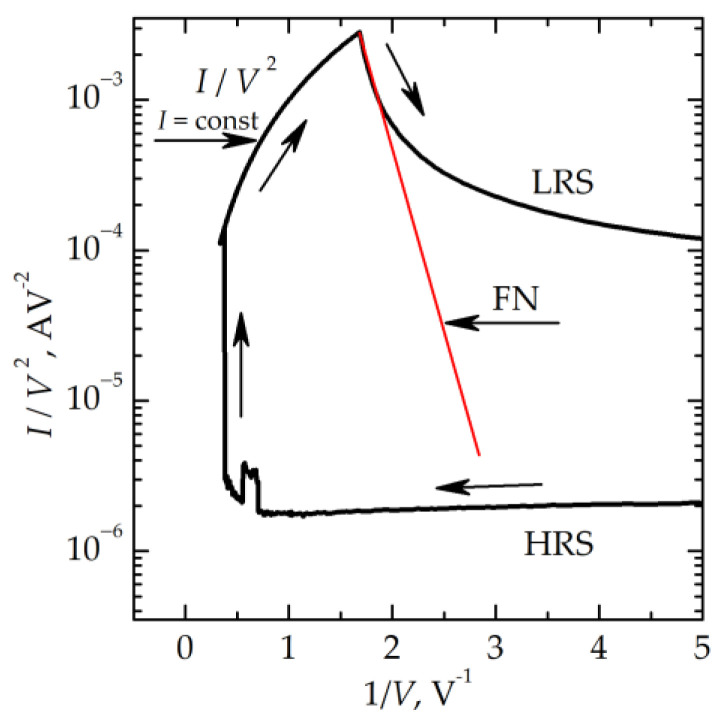
*I–V* curves of the memristive device during switching from an HRS to an LRS measured at 77 K and plotted in the Fowler–Nordheim scales. The directions of the voltage sweep are shown by arrows.

Note also that the *I–V* curves of memristive devices can be fitted using the Fowler–Nordheim (FN) law in a certain voltage range marked with a red straight line in Figure 9.

The effective electron mass m* can be estimated from the slope of the respective part of the *I–V* curve in the Fowler–Nordheim coordinates as 1.6 *m*_0_ (*m*_0_ is the free electron mass). This value is much higher than the effective electron mass in silicon oxide (m* ≈ 0.4∙*m_0_*) [16]. On the other hand, the Fowler–Nordheim dependence *I*(*V*) also points to the trap-assisted tunneling as the predominant electron transport mechanism in the filament since the logarithm of electron tunneling probability between the traps should be proportional to 1/*V* [19]. All the above points to trap-assisted tunneling as the predominant mechanism of electron transport through the filaments.

One can note that the number of points to fit within the FN parts of the *I–V* curves is rather small. Nevertheless, we think that considering the FN fit of the respective parts can be justified due to the following reasons. First, the shapes of the *I–V* curves are quite typical for the cases when the field electron emission in the MIM stacks is observed (see e.g., [20]). Typically, the FN parts are observed at a higher bias, and the bias range, for which the FN-type *I*(*V*) dependence is observed, is narrow, inevitably because of a rapid (super-exponential) growth of *I* with increasing bias *V* in the FN regime. When studying the *I–V* curves of the MIM stacks, the upper limit for the further increase of the bias is set by the electrical breakdown of the insulator (unlike the situation, e.g., when studying the field electron emission in a vacuum when the upper limit for the bias is set by the self-ion emission phenomenon).

In turn, it is necessary to note the importance of taking into account the change in electron transport during resistive switching in modeling the behavior of memristive devices, as was performed in Ref. [21], which presents a physical model describing resistive switching taking into account the trap-assisted transition between the Schottky emission and the Fowler–Nordheim tunneling, and successfully reproducing the memristive behaviors occurring on the interface between Bi_2_S_3_ nano-networks and F-doped SnO_2_. The research carried out by the authors of [21] made it possible not only to reveal several features of the memristive interface including the distribution nature of the traps, the barrier height/thickness, and so on, but also to provide a foundation on which the real interfacial memristor can be quantitatively modeled.

Quantitatively, the trap-assisted tunneling mechanism was confirmed by the *C–V* and *G–V* measurements. After switching, the extended nonlinear regions were observed on the *C–V* and *G–V* curves. From these data, the concentration of traps and the electron transport mechanism in the filaments can be evaluated.

**Figure 10:**
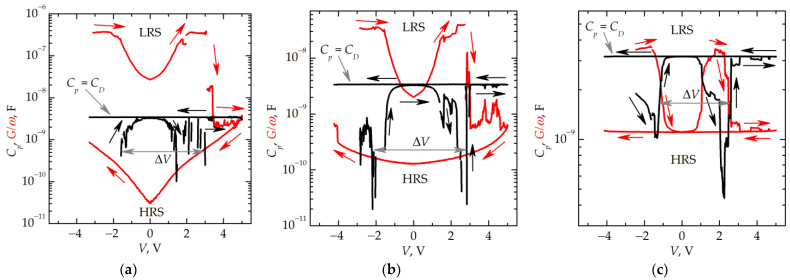
*C–V* and *G–V* curves of the memristive device in LRS and HRS obtained at 77 K and different *f_ss_*: (**a**) 10 kHz, (**b**) 100 kHz, and (**c**) 1 MHz. The data were obtained by applying a triangular voltage sweep, varying from −5 to +5 V and vice versa.

The *C–V* and *G–V* curves measured at 77 K for different frequencies of the small test signal *f_ss_* are shown in Figure 10. The *C–V* and *G–V* curves measured at different values of *f_ss_* agree with each other qualitatively. Initially, *C_p_* decreases abruptly when applying a negative voltage while the *G/ω* values increase, and *G/ω* >> *C_p_*. This corresponds to the formation of filaments in the insulator and the switching to an LRS. At a further increase in the voltage up to *V* = 0, the conductivity of the filaments (owing to the electrons injected from the top electrode) decreases, and the capacitance increases. At *V* = 0 V, *C_p_* becomes equal to the capacitance of the insulator *C_D_*. At a further increase in the voltage (*V* > 0), the conductivity increases again due to the injection of electrons from the TiN bottom electrode, while the capacitance decreases accordingly due to an increase in the Ohmic leakage, so *G/ω* >> *C_p_* again. The application of a voltage of +3–+4 V leads to the rupture of filaments and switching to an HRS. In an HRS, the value of *C_p_* does not depend on the voltage applied and is equal to *C_D_* whereas the value of *G/ω* is small, and *G/ω* << *C_p_* at a sufficiently high *f_ss_* (Figure 10b,c). During the backward voltage sweep from +5 V down to –5 V, there is no switching to an LRS. This can be explained by the charge accumulation inside the insulator, which screens the external electric field applied. Also, note that, at *f_ss_* = 1 MHz, the conductivity does not depend on voltage in the course of the reverse voltage sweep (Figure 10c). A decrease in *f_ss_* leads to a stronger dependence of *G/ω* on *V* (Figure 10a,b). This indicates the filaments not to be destroyed completely in an HRS.

One can estimate the volume density of the charge carriers trapped during switching *N* from the area inside the hysteresis loop in the *G/ω*–*V* curve (Figure 10)

(1) $$N=\frac{\oint \frac{G}{\omega }dV}{q}\frac{1}{S\cdot d},$$

where *q* is the electron charge, *S* is the area of the top electrode, and *d* is the thickness of the insulator. Its density depends on *f_ss_* and is found to be (7.6 ± 0.6) × 10^20^, (5.2 ± 0.5) × 10^19^, and (1.2 ± 0.2) × 10^19^ cm^−3^ for *f_ss_* = 10, 100, and 1000 kHz, respectively.

**Figure 11:**
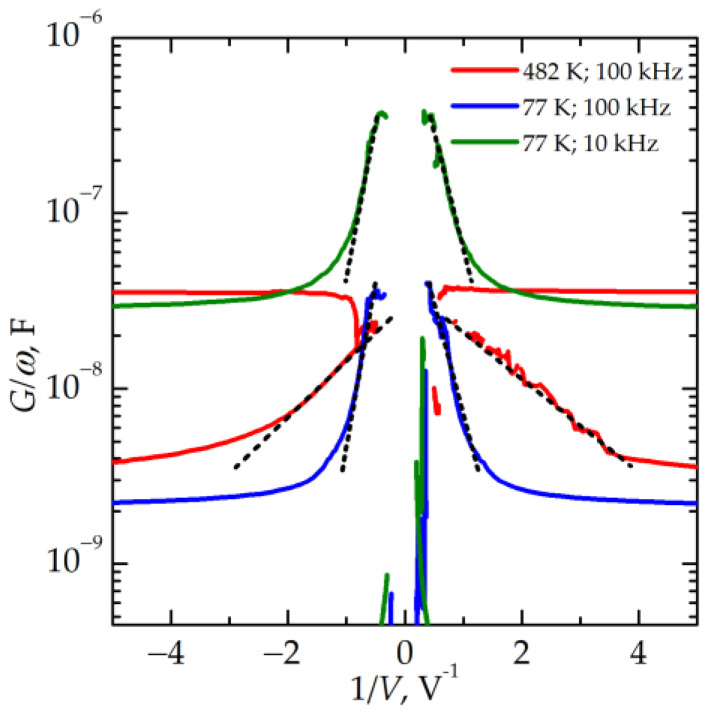
Dependencies of conductivity *G/ω* on the reciprocal voltage 1/*V* in LRS measured at different temperatures and *f_ss_*.

From the *C–V* curves, one can estimate the effective density of electron traps *N_t_* occupied by the charge carriers during switching to an LRS:

*N_t_* = *C_D_*∙Δ*V*/*q*∙*S*∙*d*, (2)

where ∆*V* is the parameter shown in Figure 10, which equals 4–5 V regardless of the value of *f_ss_*. The obtained value of *N_t_* ≈ 8× 10^18^ cm^−3^ is close to the concentration of traps in silicon oxide originating from the Si–Si bonds [22]. At the same time, these traps are nonuniformly distributed in the oxide film with a much higher local concentration in the filaments formed at the places where the electric fields are concentrated. It should be noted that the measurements of a static capacitance at temperatures up to 600 K do not reveal any increase in capacitance due to the ion migration polarization. So far, one can conclude that the electron transport mechanism along the filaments in the memristive devices studied in the present work is the electron tunneling through the defects built inside the filaments. This is confirmed by the dependences of conductivity on the reciprocal voltage in an LRS, which can be fitted by straight lines in the lg*G/*ω–1/*V* scales (Figure 11).

**Figure 12:**
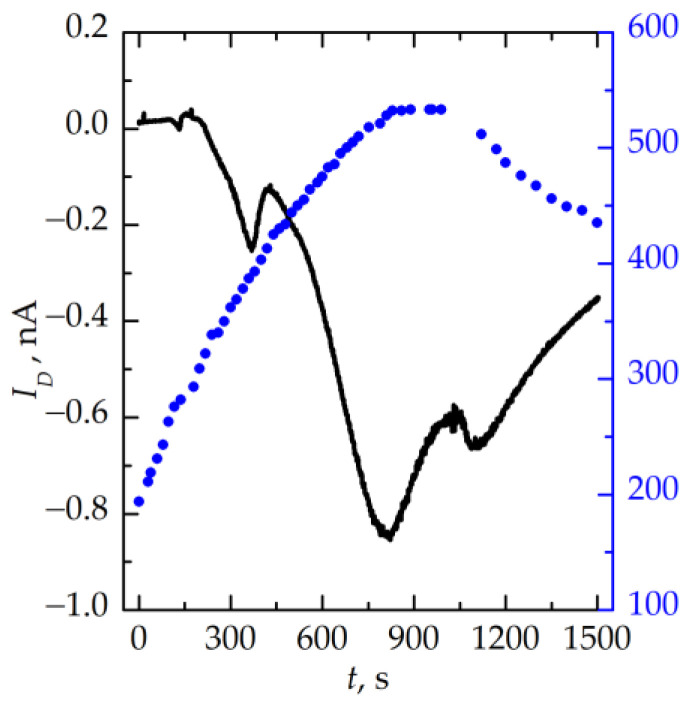
Time dependence of depolarization currents and temperature of the memristive device in HRS.

The total density of polarizable charges in the memristive device and their activation energies were determined from the measurements of the depolarization current *I_D_* in an HRS [23]. An example of a depolarization curve is shown in Figure 12. The data presented are obtained after the polarization of the stack at a constant voltage of +1 V and the temperature of 550 K followed by heating from 170 up to 550 K and cooling down to 430 K at zero voltage.

These data allow for determining the surface density of depolarized charges as an integral over the area under the depolarization current curve [23]. The value of the surface density of the depolarized charges of 5 × 10^14^ cm^−2^ is obtained, which corresponds to ~4 × 10^20^ cm^−3^ in the bulk of the insulator. Several parts of the depolarization current curves can be fitted by the straight lines in Arrhenius scales that allows for determining the activation energies of carrier traps. The values of *E_a_* of 0.33 ± 0.01, 0.38 ± 0.01, and 0.69 ± 0.08 eV are obtained. The value of *E_a_* = 0.69 ± 0.08 eV can be ascribed to the electron trap associated with the Si–Si bond [24].

**Figure 13:**
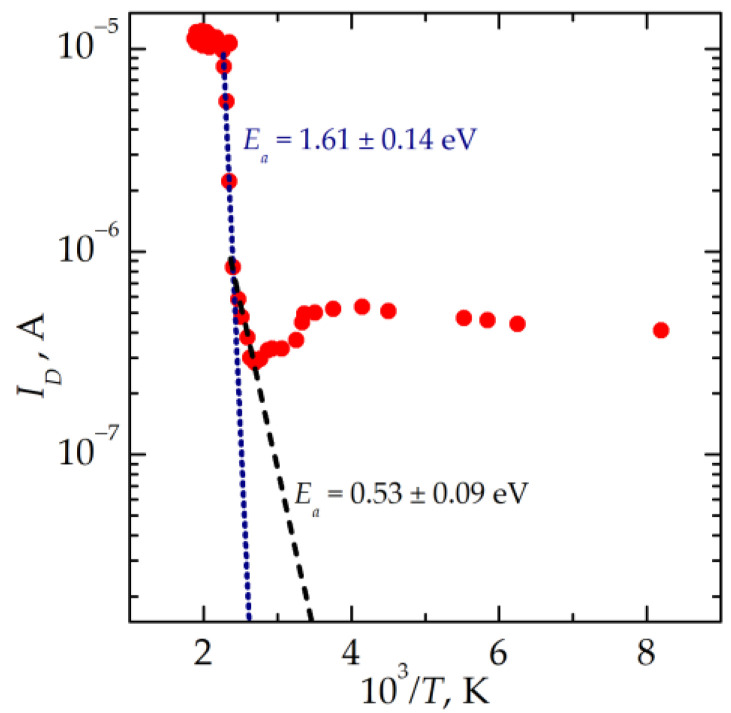
The depolarization current curve of the memristive stack in LRS in the Arrhenius scales. Experimental data are shown by red dots; approximating lines with different slopes are shown by dashed and dotted lines of different colors.

The depolarization current curve of the memristive device in an LRS is shown in Figure 13. Two values of the activation energy of the traps are found in an LRS. These values are 0.53 ± 0.09 and 1.61 ± 0.14 eV. The latter value is close to the energy of thermal ionization from the Si–Si amphoteric trap (1.5–1.7 eV [22,25]). The traps with smaller activation energies can be ascribed to the O_3_ = Si–Si = O_3_ species.

Let us now discuss how the obtained data correlate with the mechanism of the formation and destruction of conductive filaments in similar devices with a relatively thicker SiO*_x_* layer obtained by magnetron sputtering [26]. That mechanism was based on the migration of oxygen ions (O^2−^) leaving the oxygen vacancies (Si-Si bonds) that form the filament in SiO*_x_* film, the accumulation of oxygen ions in the bottom TiN electrode, and their return to the filament region with partial oxidation of the latter. It is obvious that all these processes should also take place in the devices under study; however, the small thickness and defect composition of the film obtained by the low-temperature method determine the specifics of these processes even in the as-deposited state, which is initially conductive. The structure of filaments can also change dramatically upon an additional thermal treatment, which manifests itself in the observed regularities of electron transport. Although the general dependencies, consisting of the semiconducting-like temperature dependence of conductivity in a low-resistance state and the activation nature of current in a high-resistance state, are reproduced, these studies allow us to look deeper inside the nature of electronic processes in Si-based filaments, which are reflected in the electrical characteristics of new CMOS-compatible memristive devices.

## 4. Conclusions

To summarize, the electrical characteristics and resistive switching properties of CMOS-compatible SiO*_x_*-based memristive devices are reported. In the initial state and before the thermal treatment, the device stacks do not require electroforming, but cannot store the recorded state. This is attributed to the impact of conductive defects formed during the fabrication of the stacks. After the thermal treatment, the devices exhibit bipolar resistive switching with a gradual transition between multiple resistances typical of synaptic behavior. This synaptic switching causes a change in the dielectric losses in the devices (and hence in the resistive switching characteristics) in the low frequency range (<10^5^ Hz) only. When switching to the high-resistance state at elevated temperatures (482 K), the filaments do not break completely. At low temperatures (77 K), no dielectric losses associated with the Ohmic leakages (through the filaments) are found. The mechanisms of current transport in different resistive states of the memristive device are suggested: the Schottky or the Poole–Frenkel mechanism in the high resistance state and trap-assisted tunneling in the low resistance state. The values of the density and activation energies of traps in the SiO*_x_* film are determined and are similar to those of typical defects constituting conductive filaments in silicon oxide.

## Data Availability

The data that support the findings of this study are available within the article.
